# Molecular Targets for the Treatment of Juvenile Myelomonocytic Leukemia

**DOI:** 10.1155/2012/308252

**Published:** 2011-11-13

**Authors:** Xiaoling Liu, Himalee Sabnis, Kevin D. Bunting, Cheng-Kui Qu

**Affiliations:** ^1^Division of Hematology and Oncology, Department of Medicine, Center for Stem Cell and Regenerative Medicine, Case Comprehensive Cancer Center, Case Western Reserve University, Cleveland, OH 44106, USA; ^2^Aflac Cancer Center of Children's Health Care of Atlanta and, Department of Pediatrics, Emory University, Atlanta, GA 30322, USA

## Abstract

Significant advances in our understanding of the genetic defects and the pathogenesis of juvenile myelomonocytic leukemia (JMML) have been achieved in the last several years. The information gathered tremendously helps us in designing molecular targeted therapies for this otherwise fatal disease. Various approaches are being investigated to target defective pathways/molecules in this disease. However, effective therapy is still lacking. Development of specific target-based drugs for JMML remains a big challenge and represents a promising direction in this field.

## 1. Juvenile Myelomonocytic Leukemia (JMML) and Current Clinical Standard of Care

Juvenile myelomonocytic leukemia (JMML) is a rare hematologic malignancy of early childhood with high mortality. It represents 2% to 3% of all pediatric leukemias [1, 2], and its incidence is approximately 0.6 per million children per year [3]. Clinically, patients often present with pallor, failure to thrive, decreased appetite, irritability, dry cough, tachypnea, skin rashes, and diarrhea and are found to have lymphadenopathy and hepatosplenomegaly on examination [4–8]. JMML is characterized by leukocytosis with prominent monocytosis, thrombocytopenia, elevation of fetal hemoglobin (HbF), and hypersensitivity of hematopoietic progenitors to granulocyte-macrophage colony-stimulating factor (GM-CSF) [4–8]. 

Prior to the revision in 2008, JMML was diagnosed based on the following criteria: presence of peripheral blood monocytosis (>1000/*μ*L); less than 20% blasts in the bone marrow; absence of Philadelphia (Ph) chromosome or BCR-ABL fusion gene AND at least two of the following criteria: increased HbF levels; presence of immature myeloid precursors in the peripheral blood; white blood cell count >10,000/*μ*L; GM-CSF hypersensitivity of myeloid progenitors *in vitro* [5, 9]. In 2008, the JMML diagnostic criteria were revised to account for the molecular genetic abnormalities that were identified in this disease [5]. 

The natural course of JMML is rapidly fatal with 80% of patients surviving less than three years [10]. Low platelet count, age at diagnosis older than 2 years, and high HbF percentage have been shown to correlate with poor outcome [11]. Allogeneic hematopoietic stem cell transplantation (HSCT) is currently the only curative treatment for JMML, but controversy exists in identifying the patients that need to proceed to transplant immediately versus those that can be observed for a longer time. Patients with Noonan syndrome often develop a JMML-like myeloproliferative disorder that may resolve spontaneously within one year of presentation [12]. While awaiting transplant, most patients receive chemotherapy, and most clinicians will use cytarabine-based acute myeloid leukemia-like therapy [10, 13]. Identification of gene mutations in the RAS-MAPK pathway has increased interest in development of drugs that can specifically affect molecular targets. For more detailed review of genetic mutations in JMML and approaches to therapy, please refer to Dr. Loh's recent article [14]. Here, we shall proceed to briefly discuss molecular defects in JMML with focus on potential drug targets.

## 2. Identification of Genetic Mutations in JMML

The molecular defects in JMML result in deregulated signaling through the RAS pathway [15–17]. These mutations are mutually exclusive which highlights the major functional role of the RAS pathway activation in JMML pathophysiology and disease progression. The specific genes implicated in JMML are summarized in Table 1. 

### 2.1. *PTPN11*

Somatic mutations within *PTPN11*, which encodes protein tyrosine phosphatase SHP-2, have been found in 35% of JMML patients [18, 19]. *PTPN11* mutations were also associated with poor prognosis for survival. Mutation in *PTPN11* was the only unfavorable factor for relapse after hematopoietic stem cell transplantation [20]. SHP-2 contains 2 Src homology 2 domains (N-SH2 and C-SH2) at the amino terminus and a phosphatase domain at the carboxy terminus [21–25]. It is involved in a variety of signaling pathways, especially the RAS/MAPK/ERK pathway [26–28]. SHP-2 is normally self-inhibited by hydrogen bonding of the backside of the N-SH2 domain loop to the deep pocket of the PTP domain [29, 30]. The self-inhibition leads to occlusion of the phosphatase catalytic site and a distortion of the pY-binding site of N-SH2. *PTPN11* mutations found in JMML are mainly localized in the N-SH2 domain. These mutations result in amino acid changes at the interface formed between N-SH2 and PTP domains, disrupting the inhibitory intramolecular interaction, leading to hyperactivation of SHP-2 catalytic activity [18, 31]. In addition, disease mutations enhance the binding of mutant SHP-2 to signaling partners [32–34]. Recent studies have shown that *PTPN11* gain-of-function mutations induce cytokine hypersensitivity in myeloid progenitors [16, 34, 35] and myeloproliferative disease with some similarity to JMML in mice [32, 36–38], establishing the causal role of *PTPN11* mutations in the pathogenesis of JMML. It is evident that increased signal transduction along SHP-2's pathways leads to aberrant hematopoietic cell proliferation and differentiation. SHP-2 may, thus, be an ideal target of mechanism-based therapeutics for this disease.

### 2.2. *RAS*

The *RAS* subfamily includes three members: *HRAS*, *KRAS*, and *NRAS*. Twenty-five percent of JMML patients were found to have a somatic *NRAS* or *KRAS* point mutation [20, 39]. Flotho et al. analyzed 36 children with JMML. *RAS* mutations were detected in 6 cases. Two children had a mutation in codon 12 of *NRAS*, 3 children in codon 13 of *NRAS*, and 1 child in codon 13 of *KRAS*. No mutation in *HRAS* codons 12, 13, or 61 was found [40]. De Filippi et al. reported a 38G > A (G13D) mutation in the *NRAS *gene in all types of cells checked in a male infant who was diagnosed with JMML [41]. This case suggests that constitutively active mutations of *NRAS* may be responsible for the development of JMML in children [41].

### 2.3. *NF1*

In 1994, Shannon et al. demonstrated loss of the wild-type *NF1* allele in the diseased bone marrow of children with JMML affected by neurofibromatosis type 1 (NF1) [42]. The protein product of *NF1*, neurofibromin (NF1), contains a GTPase-activating protein- (GAP-) related domain. It inhibits RAS signaling by increasing the intrinsic GTPase activity of RAS-GTP and, thus, the generation of inactive RAS-GDP [43]. Eleven percent of JMML patients have constitutive NF1 [44] and 15% of the JMML patients without clinical signs of NF1 [39, 45]. A mitotic recombination event in JMML-initiating cells led to 17q uniparental disomy with homozygous loss of normal *NF1*, providing confirmatory evidence that the* NF1* gene is crucial for the increased incidence of JMML in NF1 patients [44]. In addition, children (but not adults) with NF1 show a 200- to 500-fold increase in the incidence of *de novo* malignant myeloid disorders, particularly JMML [46].

### 2.4. CBL

The Casitas B-cell lymphoma (CBL, c-CBL) protein is a member of the CBL family of E3 ubiquitin ligases. Loh et al. first reported that *c-CBL* mutations were detected in 27 of 159 JMML samples, and 13 of these mutations alter codon Y371 [47]. The same *c-CBL* mutation was also found in another study with a smaller cohort of JMML patients [48]. A recent study screened *CBL* mutations in 65 patients with JMML [49]. A homozygous mutation of *CBL* was found in leukemic cells of 4/65 (6%) patients. A heterozygous germ line *CBL *Y371H substitution was found in each of them and was inherited from the father in one patient. The germ line mutation represents the first hit, with somatic loss of heterozygosity being the second hit positively selected in JMML cells [49]. Individuals with germ line *CBL* mutations are at increased risk of developing JMML, which might follow an aggressive clinical course or resolve without treatment [50]. 

In addition to *PTPN11*, *RAS*, and *NF1* mutations, other mutations have been reported to occur rarely in JMML, such as additional sex combs like 1 (*AXSL1*) [51] and fms-like tyrosine kinase 3 (FLT3) [52]. Mutations in let-7 or in binding sites of let-7 mRNA targets lead to an upregulation of *RAS* genes in JMML. It is possible that other microRNAs known to bind to *NRAS*- or *KRAS*-UTR, or other let-7 family mi-RNAs may play a role in the development of JMML [53]. However, mutations which are reported to play a major role in myeloproliferative neoplasms, such as ten-eleven translocation-2 (*TET2*), runt-related transcription factor 1 isoform (*RUNX1*), janus kinase 2 (*JAK2*) V617F [54], and Soc-2 suppressor of clear homolog* SHOC2 *[55] are not involved in JMML.

## 3. Chromosomal Aberrations

Some chromosome abnormalities were found in JMML. The most common chromosome abnormalities in JMML patients are monosomy 7 or deletion 7q (-7/del(7q)) [56]. In addition, there are some case reports for other chromosomal aberrations. For example, a 11-month-old boy with JMML had deletion 5q as the sole clonal chromosome abnormality [57]. Another JMML patient had a chromosomal translocation at t(1;5) [58]. Also, leukemic cells in a JMML patient harbored a 46,XX,der(12)t(3;12) (q21~22;p13.33) karyotype and subsequently developed partial trisomy of 3q [59]. However, at this time specific genes associated with these breakpoints are not yet identified, and; thus, the relevance of these chromosomal aberrations remains to be determined. 

## 4. Recent Experimental Therapy for JMML

**Figure 1:**
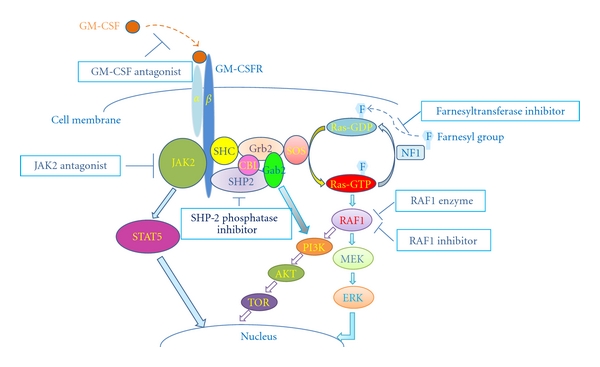


The recent focus in JMML has concentrated on using the information gained from knowledge of these molecular defects in order to design targeted drug therapy. Animal models, especially mouse models of the disease, are commonly used to test molecularly targeted agents. Since RAS hyperactivation is very important in the pathophysiology of JMML, agents designed to decrease RAS activity are being evaluated. There are numerous approaches that have been tested to target this pathway (Figure 1).

### 4.1. RAF1 Enzyme

RAF1 is a MAP kinase (MAP3K) that functions downstream of the RAS subfamily of membrane-associated GTPases to which it binds directly and plays an important role in the MAPK/ERK signal transduction pathway as part of a protein kinase cascade. A DNA enzyme designed to specifically cleave mRNA for RAF1, named RAF1 enzyme, was tested on JMML cells cultured both *in vitro* and in a xenograft model of JMML. When immunodeficient mice engrafted with JMML cells were treated continuously with this enzyme for 4 weeks, JMML cell numbers in the recipient murine bone marrows were profoundly reduced. No effect of the enzyme on the proliferation of normal bone marrow cells was found *in vitro*, indicating its specificity and potential safety [60].

### 4.2. RAF1 Inhibitor: BAY 43-9006

BAY 43-9006 is a low-molecular-weight agent that inhibits both the wild-type BRAF and the activated V599E mutant BRAF by binding at the active site of the kinase [61]. BAY 43-9006 can significantly inhibit tumor growth in a dose-dependent manner and has demonstrated oral *in vivo *activity in three human tumor xenograft models with mutant *KRAS *genes (*HCT116*, *MiaPaca-2*, *H460*) and one human tumor xenograft with a wild-type *KRAS *but exhibiting overexpression of growth factor receptors for epidermal growth factor (EGF) and HER 2 (SKOV-3) [62]. Based on these findings, a phase II window clinical trial is under development to evaluate response rate and acute toxicity to JMML patients [5].

### 4.3. Farnesyltransferase Inhibitor (FTI)

RAS is first activated at the cell membrane via the addition of a farnesyl group to the newly translated protein. Farnesyltransferase inhibitors (FTIs) can prevent RAS translocation to the plasma membrane, thus, leading to downregulation of RAS-activated cellular pathways, so its competitive inhibitors have been developed as a novel class of anticancer therapeutics. L-744,832 is one such farnesyltransferase inhibitor. It can inhibit HRAS prenylation in cell lines and in primary hematopoietic cells, abolish the *in vitro* growth of myeloid progenitor colonies in response to GM-CSF, and increase the amount of unprocessed HRAS in bone marrow cells. However, FTIs had no detectable effect on NRAS, and the mouse model with JMML features created by transplantation of Nf1^−/−^ fetal liver cells did not respond to L-744,832 treatment [63]. L-739,749, another kind of FTI, also has significant growth inhibitory effects *in vitro*, indicating a potential treatment for JMML [64]. Unfortunately, FTIs have modest to little activity in clinical trials when used as a single agent to treat cancers, yet recent studies show that when combined with other inhibitors, such as AKT inhibitors, FTIs do show a therapeutic potential in some cancer models [65, 66].

### 4.4. SHP-2 Phosphatase Inhibitor

The direct connection between activating mutations in *PTPN11* and JMML indicates that SHP-2 may be a useful target for mechanism-based therapeutics for this disease. It is very important to develop selective SHP-2 inhibitors. The availability of SHP-2-specific inhibitors could lead to the development of new drugs that would ultimately serve as treatments for JMML. However, development of selective SHP-2 inhibitors has been challenging as the catalytic site of SHP-2 shares a high homology with those of other tyrosine phosphatases, especially SHP-1 that plays a negative role in cytokine signaling in contrast to SHP-2 phosphatase [26–28]. Several groups have attempted to identify low molecular weight inhibitors for SHP-2 phosphatase using various approaches [67–70]. However, the inhibitors identified to date either show low or no selectivity between SHP-2 and highly related SHP-1 phosphatase. Furthermore, therapeutic effects of these inhibitors in mouse models or human JMML samples have yet to be determined. More efforts are still needed to advance this line of research. 

### 4.5. GM-CSF Antagonist: E21R

GM-CSF markedly promotes proliferation and survival of JMML cells and, thus, contributes to the aggressive nature of this malignancy [71]. Iversen et al. developed a GM-CSF analogue (E21R) that carries a single point mutation at position 21 in which glutamic acid is substituted for arginine [72]. It can effectively antagonize GM-CSF in binding experiments and in functional assays. They administrated E21R or isotonic saline to SCID/NOD mice transplanted by JMML cells or normal bone marrow cells and found that E21R reduced growth of JMML cell load in the mouse bone marrow [8]. As TNF*α* may increase the production of GM-CSF [71], E21R also synergizes with anti-TNF*α* monoclonal antibody (MoAb) cA2 in suppressing JMML cell growth. Remarkably, E21R preferentially eliminated leukemic cells [8]. These data suggest that E21R may have a therapeutic potential in JMML.

### 4.6. Inhibition of Angiogenesis

Angiogenesis is essential for growth and metastasis of solid tumors and probably also for hematological malignancies. Endostatin and PI-88, two kinds of angiogenic inhibitors, were used to treat JMML xenograft mice and resulted in a reduction of about 95% of the malignant cell load. Furthermore, it was evident that neither endostatin nor PI-88 interfered with the engraftment of normal cells [73].

### 4.7. STAT5 Activation by the RAS/RAF/MEK/ERK Pathway in JMML-Biomarker and Potential Therapeutic Target

In addition to activation of the RAS pathway in JMML, there are several studies that have also shown enhanced signal transducer and activator of transcription 5 (STAT5) activation downstream of activated RAS. In a KRAS G12D mouse model, STAT5 activation was also associated with ERK and S6K phosphorylation [74]. KRAS also led to hyper-active STAT5, AKT, and ERK pathways [75]. Furthermore, NRAS caused an adult CMML-like phenotype characterized by ERK and STAT5 activation via a GM-CSF-dependent induction mechanism [76]. In patient samples, Kotecha et al. [77] elegantly showed that both ERK and STAT5 activation are associated with human JMML, but, interestingly, it was the phosphorylated STAT5 that was prognostic in these patients, suggesting that effective suppression of STAT5 will also be an important biomarker in JMML-oriented targeted therapies. Therefore, monitoring pSTAT5 by phospho-flow cytometry shows promise for clinical application. Additionally, STAT5 can partner with the adapter protein GAB2 to provoke activation of the PI3-kinase pathway [78]. Interestingly, GAB2 is also a major partner of oncogenic SHP-2 with gain-of-function mutation D61G and is responsible for a significant contribution to the myeloproliferative disease phenotype in mice [38].

## 5. Discussions and Perspectives

The clinical therapy of JMML has significantly improved over the last 20 years. However, the low incidence of the disease has limited the capacity to perform large-scale pathophysiological studies and testing newer therapeutic strategies. JMML is a disease that only occurs in children, and drug dosage modifications are needed in children as compared to adults. All these factors limit the development of JMML treatment to some extent. Specific inhibitors for the molecular targets identified in this disease are still lacking. 

Molecular mechanisms of JMML have been elucidated in almost 85% of patients, but it is also true that a few JMML patients with Noonan syndrome can spontaneously recover without intervention [79, 80]. This means that we do not completely understand this disease, and there is much more to learn. It is indeed promising that there have been some novel agents evaluated in investigational phase II trials of JMML patients [81, 82], and there is legitimate hope that the knowledge we have gained about JMML will soon translate into more efficacious treatment modalities. Scientists and clinicians should continue to study molecular defects in JMML in a concerted effort to define novel therapeutic targets and to develop effective, less toxic, therapeutic interventions.

## Figures and Tables

**Table 1:** Summary of genetic mutations in JMML.

Gene	Site of mutation	Frequency
*PTPN11*	E76K, D61Y, D61V, E69K, A72T, A72V, E76V/G/A,	35%
*RAS*		
* NRAS*	Codons 12 and 13	25%
* KRAS*	Codon 13	
* HRAS*	No mutation in codons 12, 13, and 61 was found	
*NF1*	Loss of wild-type NF1 allele	11–15%
*CBL*	Codons 371, 380, 381, 384, 396, 398, 404, and 408.	17%
	Splice sites 1227, 1228, and 1096	
